# A Bibliometric Analysis of Fragility Fractures: Top 50

**DOI:** 10.3390/medicina57060639

**Published:** 2021-06-21

**Authors:** Nicolas Vuillemin, Hans-Christoph Pape, Pol Maria Rommens, Kurt Lippuner, Klaus-Arno Siebenrock, Marius Johann Keel, Johannes Dominik Bastian

**Affiliations:** 1 Department of Orthopaedic Surgery and Traumatology, Inselspital, Bern University Hospital, University of Bern, 3012 Bern, Switzerland; nicolas.vuillemin@insel.ch (N.V.); Klaus.Siebenrock@insel.ch (K.-A.S.); Marius.Keel@insel.ch (M.J.K.); 2 Department of Trauma, University Hospital of Zurich, 8091 Zurich, Switzerland; hans-christoph.pape@usz.ch; 3 Department of Orthopaedics and Traumatology, University Medical Center of Johannes Gutenberg-University Mainz, D 55131 Mainz, Germany; pol.rommens@unimedizin-mainz.de; 4 Department of Osteoporosis, Inselspital, Bern University Hospital, University of Bern, 3012 Bern, Switzerland; kurt.lippuner@insel.ch

**Keywords:** fragility fracture, hip, bibliometric analysis, osteoporosis

## Abstract

*Background and Objectives*: The population is aging and fragility fractures are a research topic of steadily growing importance. Therefore, a systematic bibliometric review was performed to identify the 50 most cited articles in the field of fragility fractures analyzing their qualities and characteristics. *Materials and Methods*: From the Core Collection database in the Thomson Reuters Web of Knowledge, the most influential original articles with reference to fragility fractures were identified in February 2021 using a multistep approach. Year of publication, total number of citations, average number of citations per year since year of publication, affiliation of first and senior author, geographic origin of study population, keywords, and level of evidence were of interest. *Results*: Articles were published in 26 different journals between 1997 and 2020. The number of total citations per article ranged from 12 to 129 citations. In the majority of publications, orthopedic surgeons and traumatologists (66%) accounted for the first authorship, articles mostly originated from Europe (58%) and the keyword mostly used was “hip fracture”. In total, 38% of the articles were therapeutic studies level III followed by prognostic studies level I. Only two therapeutic studies with level I could be identified. *Conclusions*: This bibliometric review shows the growing interest in fragility fractures and raises awareness that more high quality and interdisciplinary studies are needed.

## 1. Introduction

Patients with fragility fractures represent a particularly vulnerable patient group with specific demands and characteristics. Patient-centered care of this patient population requires specific medical expertise to prevent complications and to avoid loss of independence and the need for institutional care. Therefore, a multidisciplinary approach is mandatory, involving orthopedic surgeons, geriatricians, specialists in bone metabolism and pain therapy as well as physiotherapists [1].

Due to the rising numbers of fragility fractures over the last decades, they have increasingly become a key topic in clinical research with a growing number of publications reporting results of studies exploring fragility fractures. In consideration of that, the aim of the present study was to report in a bibliometric review the current research literature on fragility fractures.

We were interested to know what disciplines were active in clinical research in the field of fragility factures, in what geographical regions this research has been conducted, and what key topics were addressed in the most highly cited research articles elaborating fragility fractures.

## 2. Materials and Methods

From the Core Collection database in the Thomson Reuters Web of Knowledge, we searched for the most influential original articles with reference to fragility fractures. Fragility fractures have been defined as “a fracture that is caused by an injury that would be insufficient to fracture normal bone; the result of reduced compressive and/or torsional strength of bone.” [2]. The search was conducted on 9th of February 2021 and included all available documents. The most cited 50 articles were identified and then analyzed for their qualities and characteristics using this bibliometric review. For further interpretation, parts of the obtained data were presented in relation to estimated data for the world population provided by the United Nations (United Nations, Department of Economic and Social Affairs, Population Division (2019). World Population Prospects 2019, Online Edition. Rev. 1.) [3] and in relation to the gross domestic product (GDP) per capita (in USD) as provided by the National Accounts Sections of the United Nations Statistics Division (Basic Data Selection—amaWebClient. Available online: https://unstats.un.org/unsd/snaama/Basic (accessed on 6 April 2021)).

### 2.1. Selection Process and Eligibility Criteria

The inclusion and exclusion of articles, as well as data extraction were conducted by an orthopedic resident (N.V.) and a senior consultant orthopedic surgeon (J.D.B.), according to predefined criteria. Disagreements between investigators were solved by consensus.

The selection process was started using a title, abstract and author keywords search of the Thomson Reuters Web of Knowledge. The following search terms were used: (fragility fracture AND elderl * AND orthop *) OR (fragility fracture AND geriatr * AND orthop *) OR (fragility fracture AND older adult AND orthop *)) OR (fragility fracture AND elderl* AND surg *) OR (fragility fracture AND geriatr * AND surg *) OR (fragility fracture AND older adult AND surg *).

**Figure 1:**
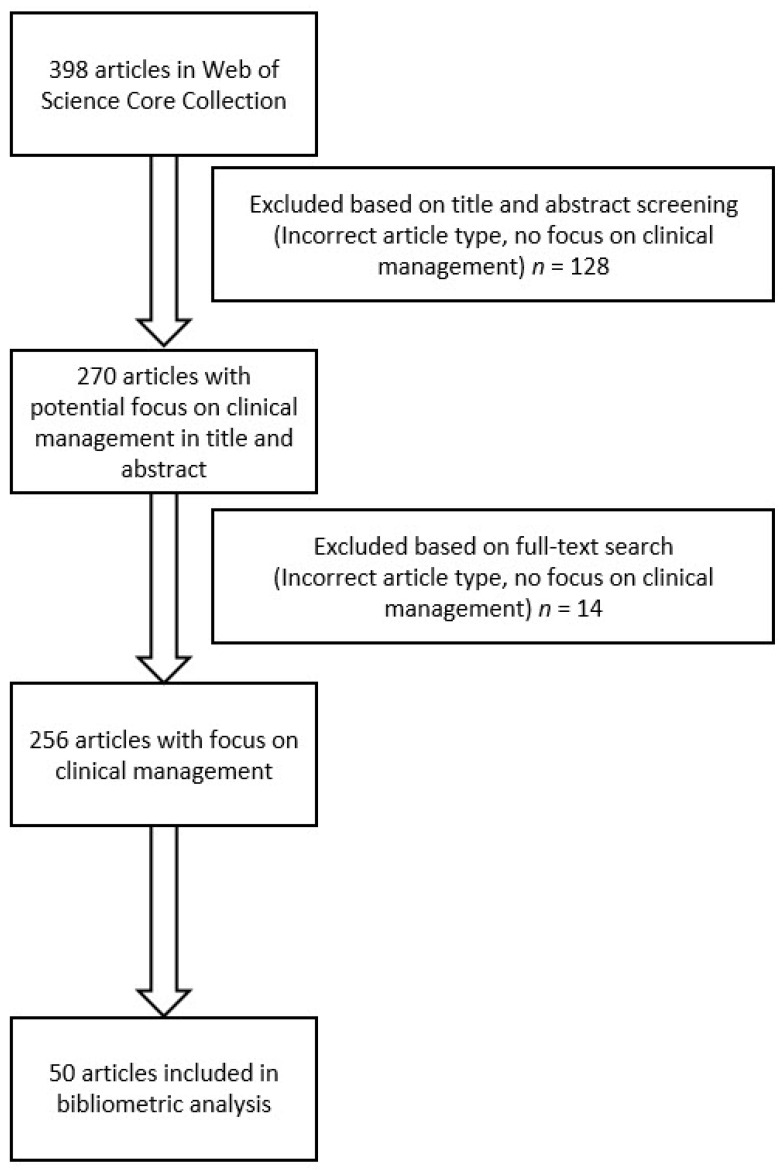
Flowchart of the selection process.

The document type was limited to original articles. Timespan was defined from 1900 to 2020. The operator “AND” was used to narrow the search. The operator “OR” was used to widen the search. The asterisk “*” was used to extend the search criteria, for example geriatr* will search for geriatrics and geriatrician. The process of inclusion and exclusion of articles is illustrated in Figure 1. The first exclusion step was performed in titles and abstracts based on either type of article (namely documents others than original articles, e.g., reviews, editorials, letter to the editor, case report, technical notes) or articles without focus on clinical management or diagnostics of fragility fractures (e.g., epidemiological studies and experimental studies, such as biomechanical studies or studies with animals). This exclusion step was redone in a second exclusion step by using full-text article search in remaining cases. For the final inclusion of identified articles, we ranked all articles according to their total citation rate; number one having the highest number of total citations. In case of an equal number of total citations, the articles were further ranked according to the average citation per year and then according to citations in 2020. For the bibliometric analysis, the 50 most cited articles were included.

### 2.2. Data Extraction and Assessment

For data analysis, information available at the Thomson Reuters Web of Knowledge on 9 February 2021 was used. For each included article, we extracted the following parameters: year of publication, total number of citations, average number of citations per year since year of publication, affiliation of first and senior author (orthopedic surgeon/traumatologist, geriatrician/internal medicine, others), geographic origin of study population, and keywords. For comparison within European countries with versus without identified articles the GDP per capita was used. Articles were classified as being either (1) therapeutic, (2) prognostic, (3) diagnostic studies, or (4) economic and decision analyses using the Journal of Bone and Joint Surgery American classification scheme [4]. The level of evidence was established according to the Journal of Bone and Joint Surgery American criteria with level I being the strongest and level V being the weakest level of evidence. Therapeutic studies were matched to an anatomic region when possible.

### 2.3. Statistical Analysis

Descriptive methods were used. All obtained data are defined as number, percentage, bar and line diagram. For analyses and plotting of diagrams Microsoft Excel, 2016 and the online tool Infogram Version 2.0.2 Available online: https://infogram.com (accessed on 2 April 2021) were used. For comparison of the GDP per capita within European countries a Mann–Whitney test was performed using GraphPad Prism (Version 9.0, GraphPad Software, San Diego, CA, USA); level of significance *p* < 0.05. For the visualization of the “fragility fracture homunculus” AutoCAD by AutoDesk, 2021, was used.

## 3. Results

**Figure 2:**
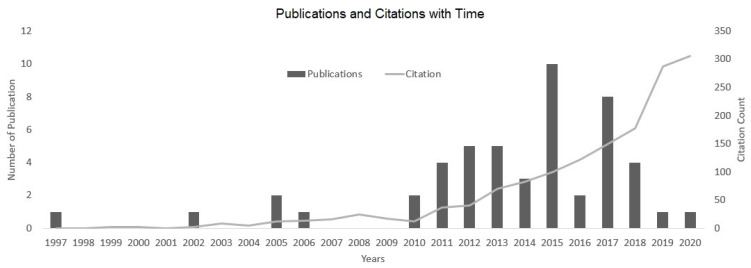
Publication and citation of identified articles with time.

The 50 most cited articles [5,6,7,8,9,10,11,12,13,14,15,16,17,18,19,20,21,22,23,24,25,26,27,28,29,30,31,32,33,34,35,36,37,38,39,40,41,42,43,44,45,46,47,48,49,50,51,52,53,54] were published between 1997 and 2020. All 50 articles were in English language. The year with the highest number of articles was 2015 (*n* = 10). In several years, 2009 and before (2008, 2007, 2004, 2003, 2001 to 1998) no article among the most 50 cited ones was published (Figure 2).

The number of total citations per article ranged from 12 to 129 citations, with a mean of 30 citations per article. The oldest study was reported in 1997 by Berlemann et al. [7]. The youngest study was published by Catellani et al. in 2020 [11]. The average citation per year was four with a deviation from 1 to 14 average citations per year. The citations in 2020 ranged from 0 to 37 citations, with an average of six citations. The 10 most cited articles are listed in Table 1.

According to the amount of total citations in descending order with authorship, title, journal, and year of publication and average citations per year. Scientists who were first author of more than one among the 50 most cited articles are listed in Table 2.

Overall, three authors published 2 of the 50 most cited articles as a fist author, and five authors published two articles as senior author. The distribution of first authors’ specialties in relation to their contribution is illustrated in Table 3.

In the majority of publications, orthopedic surgeons and traumatologists (66%) accounted for the first authorship, followed by geriatricians/internal medicine (18%) and others (16%). Orthopedic surgeons and traumatologists, being first author, published together with senior authors being almost always orthopedic surgeons and traumatologists (94%) followed by geriatricians/internal medicine and others. Geriatricians/internal medicine published with senior authors being geriatricians/internal medicine (78%) and with orthopedic surgeons and traumatologists (22%). In the case that “others” were first authors, they mainly published with others (63%) followed by orthopedic surgeons and traumatologists (38%) and none with geriatricians/internal medicine.

Articles were published in 26 different journals. Journals with more than one article are listed in Table 4.

Injury—International Journal of the Care of the Injured published the most articles (*n* = 6), equal with Osteoporosis International (*n* = 6). In total, nine journals were identified with more than one article. Those nine journals published 66% (*n* = 33) of the reviewed articles.

**Figure 3:**
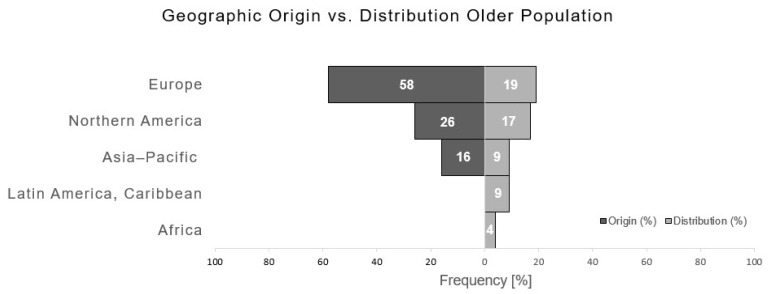
Geographic origin of publications in relation to proportion of older population.

Identified articles mostly originated from Europe (58%), followed by Northern America (26%), Asia and Pacific (16%). The analysis of the distribution of publications in relation to their geographic origin is opposed to the estimated amount of the older population (at least 65 years of age) within those different continents in Figure 3. The highest proportion of the older population is noted in Europe (19%), followed by Northern America (17%), Asia–Pacific (9%), Latin America and Caribbean (9%), and Africa (4%).

**Figure 4:**
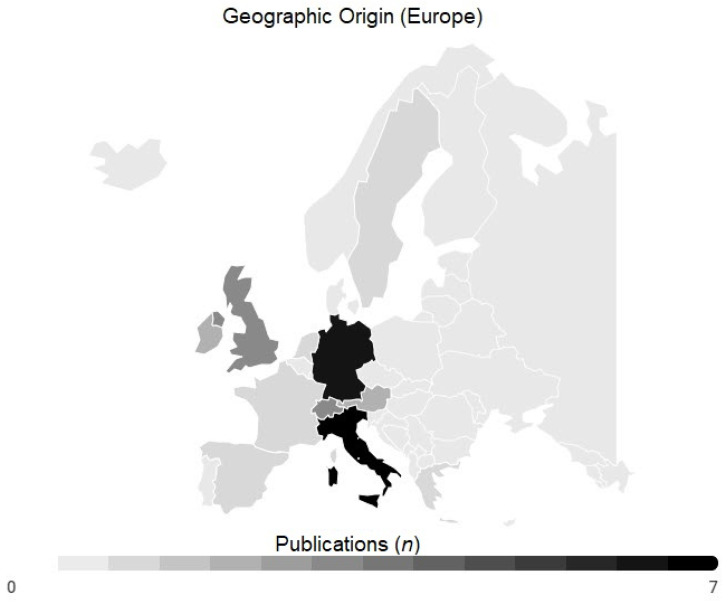
Geographic origin of articles within Europe. With minimum of zero and maximum of seven articles.

Within Europe, most publications were from Italy (*n* = 7), followed by Germany (*n* = 6), United Kingdom (*n* = 3) and Switzerland (*n* = 3), Austria (*n* = 2), Ireland (*n* = 2); Spain, France, Greece, the Netherlands, and Sweden reported one article each (Figure 4).

**Figure 5:**
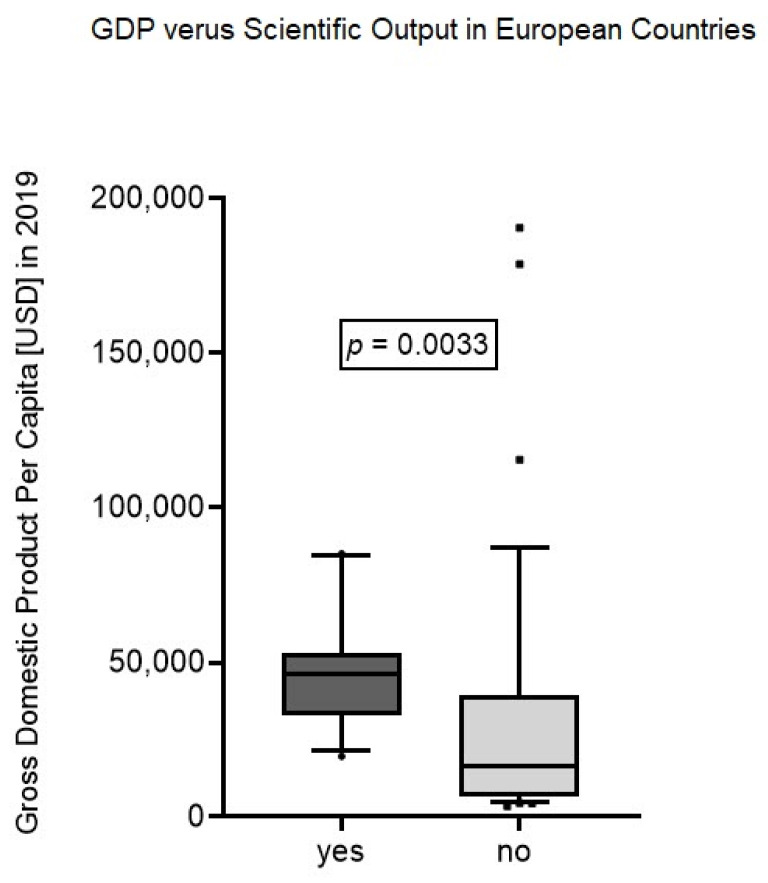
GDP versus scientific output in European countries.

In Europe, the economic power represented by the GDP was statistically significantly higher (*p* < 0.0033) in countries with a scientific output identified in this bibliometric review (*n* = 11; median GDP: USD 46232; range: 19,604–85,135) compared to remaining countries without any (*n* = 36; median GDP: USD 16.303; range: 3496–190,532) (Figure 5).

**Figure 6:**
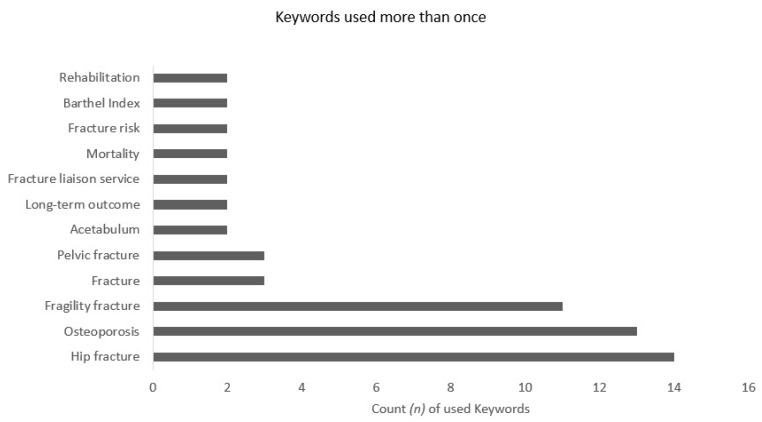
Keywords used more than once.

The analysis of keywords showed that in total 120 different keywords were used to describe the studies within the most cited 50 articles. The most frequently used keyword was “hip fracture” (used in 14 of the 50 most cited articles), followed by “osteoporosis” (13), “fragility fracture” (11), “fracture” (3), “pelvic fracture” (3); all keywords used more than once are depicted in Figure 6.

**Figure 7:**
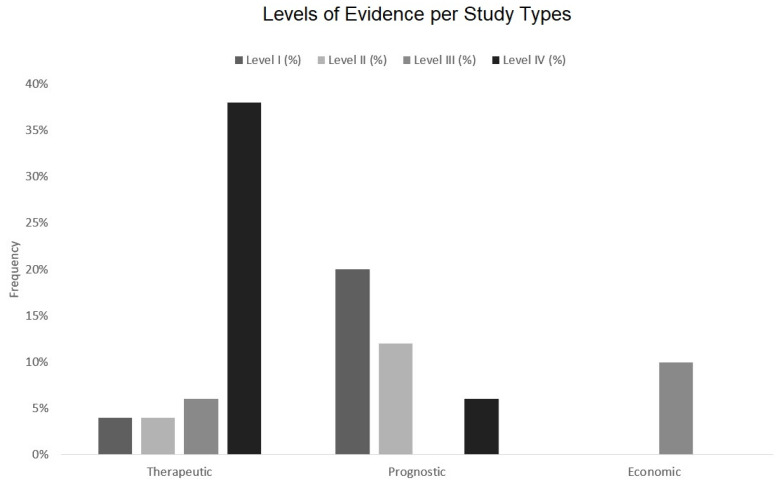
Level of evidence in the categories of therapeutic, prognostic, and economic studies.

The further analysis of study types showed the majority of articles reporting about therapeutic studies (52%), followed by prognostic studies (38%). In total, 10% of the articles reported about economic and decision analyses; no articles with diagnostic studies were identified. The levels of evidence within these study categories are presented in Figure 7. In detail, the distribution of levels of evidence were as follows for:

- Therapeutic studies *n* = 2 (4%) with level I, *n* = 2 (4%) with level II, *n* = 3 (6%) with level III, *n* = 19 (38%) with level IV.
- Prognostic *n* = 10 (20%) with level I, *n* = 6 (12%) with level II, *n* = 3 (6%) with level IV.
- Economic and decision analyses studies *n* = 5 (10%) with level III.

Out of 26 therapeutic studies, 24 focused on a specific anatomic region. For two therapeutic studies, a match to an anatomic region was not possible, due to the focus on clinical outcome of new implemented clinical pathways for all kinds of fragility fractures [12,17].

**Figure 8:**
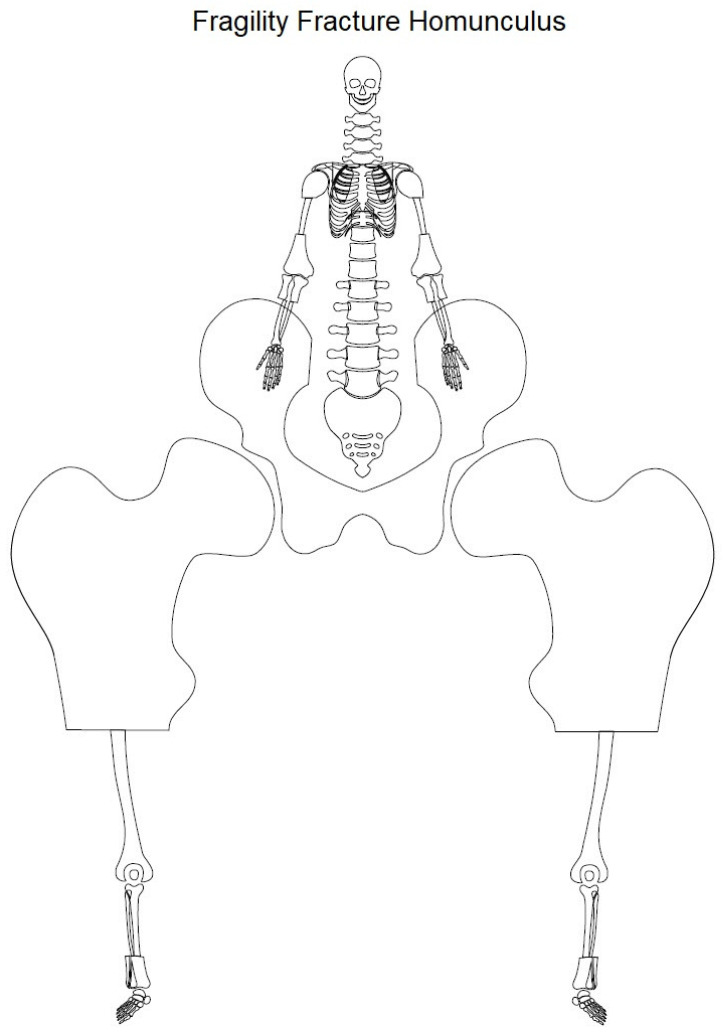
Schematic drawing illustrating the “fragility fracture homunculus” indicating different focusses of therapeutic studies represented with disproportionate sizes of the anatomical sites being reported. The anatomical sites were enlarged by the following equalization: Focus (*n*) + 1. Hip joint (*n* = 14), pelvis (*n* = 5), spine (*n* = 3), ankle (*n* = 3), distal femur (*n* = 2), elbow (*n* = 2), shoulder (*n* = 2).

Out of the 24 studies the anatomical region focused on the most was hip joint including acetabulum, femoral head, femoral neck, pertrochanteric region (*n* = 13), followed by pelvis (*n* = 4), spine (*n* = 2), ankle (*n* = 2), distal femur (*n* = 1), elbow (*n* = 1), shoulder (*n* = 1) (Figure 8).

## 4. Discussion

The aging population leads to a rapid growth of fragility fractures. Patients with fragility fractures have special needs and their physical condition differs strongly from that of younger patients, leading to new challenges for healthcare professionals [55,56]. Accordingly, the presented bibliometric review was performed to report about the existing literature on the topic of fragility fractures. This bibliometric study demonstrates that research on fragility fractures is a growing field in current clinical research. With a closer look to the development of the citations from 2010 to 2020, the number of citations raised by a factor of 26. The increase of the citations follows the trend of the growing incidence of fragility fractures, e.g., as described by Kannus et al. [55].

The stringent analysis of authorship, origin and main subject of these articles show interesting results. Therapeutic studies find key interest; however, with a low level of evidence, as in the majority a type 4 level of evidence was identified. Only a small minority among the top cited articles are randomized controlled trials. Investigating the outcome of fragility fractures, prospective studies are highly cited. The analysis concerning the field of the authors shows that orthopedic surgeons and traumatologists are the group with the most articles as first authors. With a closer look to distribution on first author and senior author, a collaboration of orthopedic surgeons/traumatologist with geriatricians/internal medicine or “others” was an infrequent finding. As a result, a well-balanced interdisciplinary team of specialists is a rare constellation, indicating the need and potential for interdisciplinary research in the future [42]. The predominance of orthopedic surgeons and traumatologists is probably related to the topic that was addressed in this bibliometric review. Many studies focus on therapeutic options of fragility fractures and prognostic factors.

The analysis of keywords revealed that “hip fracture” was the most used term in highly cited articles. Apparently, current research interest is focused on hip fractures correlating with the circumstance that hip fracture is among the most frequent operating room procedures [57]. This indicates the potential for further research areas; studies should not be limited to the hip joint. Alternatively, other regions (e.g., spinal column, pelvis, shoulder) or the prevention of fragility fractures might be in the front in future. Further, none of the articles discuss the challenges of periprosthetic fractures as it seems to have a rising incidence [58] and they are associated to fragility fractures [59]. The search strategy with the focus on fragility fractures might have neglected the periprosthetic fractures. More than the half of the cited articles were conducted in Europe, and only a minority in other geographical regions. Europe also accounts for the highest proportion of older population leading to a rising incidence for fragility fractures [60]. Those circumstances might raise the awareness of the investigators. Within Europe all articles are from Western European countries. A limited economic strength might account for that finding, as the gross domestic product per capita was significantly higher in those countries with a high amount of publications.

A limitation might be that publications not indexed within Web of Science Core Collection were not included or the search strategy or the language might have limited the number of retrieved articles. The citation count might be a measure of delay such as the study per se. A further limitation might be that in the analysis of research original articles only, but review articles were not included. An article-based, self-citation analysis was not performed, only the total number of citations was presented in this study. Criticizing an article comes with the need to cite it. This brings up the hypothesis that citations are not a sole indicator for quality [61]. The older an article is the longer it can gather citations, which could question the significance of older articles in the current literature [62]. The status on basic research and risk factors in the field of fragility fractures is not represented in this study due to the focus on clinical management in the selection process. With the focus on clinical management and by the selection of “search terms” rheumatologist, endocrinologist, and other specialized bone metabolism units as well as studies on pharmacological treatments might be under-represented.

A strength of the study is the accessibility of the data without advanced statistical methods to provide understanding of current research topics of eminent literature in a relevant global challenge for healthcare professionals. Scientists find their most important published work, amongst their most cited [63] showing that citation rates are a good tool to evaluate the impact of an article in a certain field.

## 5. Conclusions

Fragility fractures are a research topic of growing interest. Europe and the key topic (hip fracture) were key drivers in the research concerning fragility fractures. The results should encourage all disciplines to undertake more interprofessional research, leading to teams that are more balanced with a wider spectrum of interest and know how. Further, more high-quality research must be promoted especially in the form of randomized controlled studies. With the rising numbers of older people and the rising incidence of fragility fractures worldwide, the numbers of articles in the field of fragility fractures will rise simultaneously. Our bibliometric review acknowledges recent research but raises awareness that timely continuation and optimization for research in fragility fractures is needed.

## Figures and Tables

**Table 1:** List of the identified 10 most cited articles in the field of fragility fractures according to the amount of total citations with average citations per year.

N	Title	Total (*n*)	Average/Year
1	Rommens, P. M., Hofmann, A. Comprehensive classification of fragility fractures of the pelvic ring: Recommendations for surgical treatment. Injury-International Journal of the Care of the Injured 2013; 44(12): 1733–1744	129	14.3
2	Chevalley T, Hoffmeyer P, Bonjour JP, Rizzoli R. An osteoporosis clinical pathway for the medical management of patients with low-trauma fracture. Osteoporosis International. 2002; 13(6)	113	5.7
3	Kates SL, Mendelson DA, Friedman SM. The Value of an Organized Fracture Program for the Elderly: Early Results. Journal of Orthopaedic Trauma. 2011; 25(4)	76	6.9
4	Kammerlander C, Gosch M, Kammerlander-Knauer U, Luger TJ, Blauth M, Roth T. Long-term functional outcome in geriatric hip fracture patients. Archives of Orthopaedic and Trauma Surgery. 2011; 131(10)	62	5.6
5	Berlemann U, Schwarzenbach O. Dens fractures in the elderly—Results of anterior screw fixation in 19 elderly patients. Acta Orthopaedica Scandinavica. 1997; 68(4)	60	2.4
6	Samelson EJ, Demissie S, Cupples LA, Zhang XC, Xu HF, Liu CT, et al. Diabetes and Deficits in Cortical Bone Density, Microarchitecture, and Bone Size: Framingham HR-pQCT Study. Journal of Bone and Mineral Research. 2018; 33(1)	53	13.3
7	Zywiel MG, Hurley RT, Perruccio AV, Hancock-Howard RL, Coyte PC, Rampersaud YR. Health Economic Implications of Perioperative Delirium in Older Patients After Surgery for a Fragility Hip Fracture. Journal of Bone and Joint Surgery-American Volume. 2015; 97A(10)	48	6.9
8	Eekman DA, van Helden SH, Huisman AM, Verhaar HJJ, Bultink IEM, Geusens PP, et al. Optimizing fracture prevention: the fracture liaison service, an observational study. Osteoporosis International. 2014; 25(2)	45	5.6
9	Jeffcoat DM, Carroll EA, Huber FG, Goldman AT, Miller AN, Lorich DG, et al. Operative Treatment of Acetabular Fractures in an Older Population Through a Limited Ilioinguinal Approach. Journal of Orthopaedic Trauma. 2012; 26(5)	44	4.4
10	Lemon M, Somayaji HS, Khaleel A, Elliott DS. Fragility fractures of the ankle—Stabilisation with an expandable calcaneotalotibial nail. Journal of Bone and Joint Surgery-British Volume. 2005; 87B(6)	42	2.5

**Table 2:** List of authors with more than one article as first author.

Author	Affiliation	Years	Articles (*n*)
Christian Kammerlander	Department of General-, Trauma- and Reconstructive Surgery University Hospital, LMU Munich Germany	2011, 2012	2
CarlaYvonne Henderson	Graduate Entry Medical School University of Limerick Ireland	2015, 2017	2
Andreas Höch	Department of Orthopaedic, Trauma and Plastic Surgery University of Leipzig Germany	2017, 2017	2

**Table 3:** Distribution of first and senior authors’ specialties (in %).

Senior Author	1st Author		
	Orthopedic Surgeons and Traumatologist (66%)	Geriatricians/Internal Medicine (18%)	Others (16%)
Orthopedic surgeons and traumatologist	94%	22%	38%
Geriatricians/Internal medicine	3%	78%	
Others	3%		63%

**Table 4:** List of journals with more than one article within the identified articles.

Journal	Articles (*n*)
Osteoporosis International	6
Injury—International Journal of the Care of the Injured	6
Journal of Orthopaedic Trauma	4
Archives of Orthopaedic and Trauma Surgery	4
Geriatric Orthopaedic Surgery and Rehabilitation	4
Bone	3
Journal of Bone and Joint Surgery—American Volume	2
Irish Journal of Medical Science	2
Aging Clinical and Experimental Research	2

## References

[1] Rommens P.M., Wagner D., Hofmann A. (2017). Fragility Fractures of the Pelvis. JBJS Rev..

[2] Gender and Women’s Health (G.W.H.) (1998). Guidelines for Preclinical Evaluation and Clinical Trials in Osteoporosis.

[3] United Nations DoEaSA (2019). Population Division World Population Prospects 2019.

[4] Wright J.G., Swiontkowski M.F., Heckman J.D. (2003). Introducing levels of evidence to the journal. J. Bone Jt. Surg. Am. Vol..

[5] Akinleye S.D., Garofolo G., Culbertson M.D., Homel P., Erez O. (2018). The Role of BMI in Hip Fracture Surgery. Geriatr. Orthop. Surg. Rehabil..

[6] Balasubramanian A., Zhang J., Chen L., Wenkert D., Daigle S.G., Grauer A., Curtis J.R. (2019). Risk of subsequent fracture after prior fracture among older women. Osteoporos. Int..

[7] Berlemann U., Schwarzenbach O. (1997). Dens fractures in the elderly—Results of anterior screw fixation in 19 elderly patients. Acta Orthop. Scand..

[8] Biber R., Singler K., Curschmann-Horter M., Wicklein S., Sieber C., Bail H.J. (2013). Implementation of a co-managed Geriatric Fracture Center reduces hospital stay and time-to-operation in elderly femoral neck fracture patients. Arch. Orthop. Trauma Surg..

[9] Brogan K., Akehurst H., Bond E., Gee C., Poole W., Shah N.N., McChesney S., Nicol S. (2016). Delay to surgery does not affect survival following osteoporotic femoral fractures. Inj. Int. J. Care Inj..

[10] Buecking B., Bohl K., Eschbach D., Bliemel C., Aigner R., Balzer-Geldsetzer M., Dodel R., Ruchholtz S., Debus F. (2015). Factors influencing the progress of mobilization in hip fracture patients during the early postsurgical period?—A prospective observational study. Arch. Gerontol. Geriatr..

[11] Catellani F., Coscione A., D’Ambrosi R., Usai L., Roscitano C., Fiorentino G. (2020). Treatment of Proximal Femoral Fragility Fractures in Patients with COVID-19 During the SARS-CoV-2 Outbreak in Northern Italy. J. Bone Jt. Surg. Am. Vol..

[12] Chevalley T., Hoffmeyer P., Bonjour J.P., Rizzoli R. (2002). An osteoporosis clinical pathway for the medical management of patients with low-trauma fracture. Osteoporos. Int..

[13] Chiari P., Forni C., Guberti M., Gazineo D., Ronzoni S., D’Alessandro F. (2017). Predictive Factors for Pressure Ulcers in an Older Adult Population Hospitalized for Hip Fractures: A Prognostic Cohort Study. PLoS ONE.

[14] Dash S.K., Panigrahi R., Palo N., Priyadarshi A., Biswal M. (2015). Fragility Hip Fractures in Elderly Patients in Bhubaneswar, India (2012–2014): A Prospective Multicenter Study of 1031 Elderly Patients. Geriatr. Orthop. Surg. Rehabil..

[15] Doshi H.K., Ramason R., Azellarasi J., Naidu G., Chan W.L.W. (2014). Orthogeriatric model for hip fracture patients in Singapore: Our early experience and initial outcomes. Arch. Orthop. Trauma Surg..

[16] Eckardt H., Egger A., Hasler R.M., Zech C.J., Vach W., Suhm N., Morgenstern M., Saxer F. (2017). Good functional outcome in patients suffering fragility fractures of the pelvis treated with percutaneous screw stabilisation: Assessment of complications and factors influencing failure. Inj. Int. J. Care Inj..

[17] Eekman D.A., van Helden S.H., Huisman A.M., Verhaar H.J.J., Bultink I.E.M., Geusens P.P., Lips P., Lems W.F. (2014). Optimizing fracture prevention: The fracture liaison service, an observational study. Osteoporos. Int..

[18] Faucett S.C., Genuario J.W., Tosteson A.N.A., Koval K.J. (2010). Is Prophylactic Fixation a Cost-Effective Method to Prevent a Future Contralateral Fragility Hip Fracture?. J. Orthop. Trauma.

[19] Garg A.X., Pouget J., Young A., Huang A., Boudville N., Hodsman A., Adachi J.D., Leslie W.D., Cadarette S.M., Lok C.E. (2012). Fracture Risk in Living Kidney Donors: A Matched Cohort Study. Am. J. Kidney Dis..

[20] Garmilla-Ezquerra P., Sanudo C., Delgado-Calle J., Perez-Nunez M.I., Sumillera M., Riancho J.A. (2015). Analysis of the Bone MicroRNome in Osteoporotic Fractures. Calcif. Tissue Int..

[21] Georgiannos D., Lampridis V., Bisbinas I. (2017). Fragility fractures of the ankle in the elderly: Open reduction and internal fixation versus tibio-talo-calcaneal nailing: Short-term results of a prospective randomized-controlled study. Inj. Int. J. Care Inj..

[22] Giannini S., Chiarello E., Cadossi M., Luciani D., Tedesco G. (2011). Prosthetic surgery in fragility osteopathy. Aging Clin. Exp. Res..

[23] Gonnelli S., Caffarelli C., Maggi S., Rossi S., Siviero P., Gandolini G., Cisari C., Rossini M., Iolascon G., Mauro G.L. (2013). The assessment of vertebral fractures in elderly women with recent hip fractures: The BREAK Study. Osteoporos. Int..

[24] Henderson C.Y., Ryan J.P. (2015). Predicting mortality following hip fracture: An analysis of comorbidities and complications. Ir. J. Med Sci..

[25] Henderson C.Y., Shanahan E., Butler A., Lenehan B., O’Connor M., Lyons D., Ryan J.P. (2017). Dedicated orthogeriatric service reduces hip fracture mortality. Ir. J. Med Sci..

[26] Hoch A., Ozkurtul O., Pieroh P., Josten C., Bohme J. (2017). Outcome and 2-Year Survival Rate in Elderly Patients With Lateral Compression Fractures of the Pelvis. Geriatr. Orthop. Surg. Rehabil..

[27] Höch A., Pieroh P., Henkelmann R., Josten C., Böhme J. (2017). In-screw polymethylmethacrylate-augmented sacroiliac screw for the treatment of fragility fractures of the pelvis: A prospective, observational study with 1-year follow-up. BMC Surg..

[28] Jeffcoat D.M., Carroll E.A., Huber F.G., Goldman A.T., Miller A.N., Lorich D.G., Helfet D.L. (2012). Operative Treatment of Acetabular Fractures in an Older Population Through a Limited Ilioinguinal Approach. J. Orthop. Trauma.

[29] Kammerlander C., Gosch M., Kammerlander-Knauer U., Luger T.J., Blauth M., Roth T. (2011). Long-term functional outcome in geriatric hip fracture patients. Arch. Orthop. Trauma Surg..

[30] Kammerlander C., Riedmuller P., Gosch M., Zegg M., Kammerlander-Knauer U., Schmid R., Roth T. (2012). Functional outcome and mortality in geriatric distal femoral fractures. Inj. Int. J. Care Inj..

[31] Karim L., Moulton J., Van Vliet M., Velie K., Robbins A., Malekipour F., Abdeen A., Ayres D., Bouxsein M.L. (2018). Bone microarchitecture, biomechanical properties, and advanced glycation end-products in the proximal femur of adults with type 2 diabetes. Bone.

[32] Kates S.L., Mendelson D.A., Friedman S.M. (2011). The Value of an Organized Fracture Program for the Elderly: Early Results. J. Orthop. Trauma.

[33] Khan S.K., Weusten A., Bonczek S., Tate A., Port A. (2013). The Best Practice Tariff helps improve management of neck of femur fractures: A completed audit loop. Br. J. Hosp. Med..

[34] Kim J.W., Herbert B., Hao J.D., Min W., Ziran B.H., Mauffrey C. (2015). Acetabular fractures in elderly patients: A comparative study of low-energy versus high-energy injuries. Int. Orthop..

[35] Lemon M., Somayaji H.S., Khaleel A., Elliott D.S. (2005). Fragility fractures of the ankle—Stabilisation with an expandable calcaneotalotibial nail. J. Bone Jt. Surg. Br. Vol..

[36] Liu S.K.K., Ho A.W.H., Wong S.H. (2017). Early surgery for Hong Kong Chinese elderly patients with hip fracture reduces short-term and long-term mortality. Hong Kong Med. J..

[37] Mansat P., Degorce H.N., Bonnevialle N., Demezon H., Fabre T., SOFCOT (2013). Total elbow arthroplasty for acute distal humeral fractures in patients over 65 years old—Results of a multicenter study in 87 patients. Orthop. Traumatol. Surg. Res..

[38] Murena L., Moretti A., Meo F., Saggioro E., Barbati G., Ratti C., Canton G. (2018). Predictors of cut-out after cephalomedullary nail fixation of pertrochanteric fractures: A retrospective study of 813 patients. Arch. Orthop. Trauma Surg..

[39] Pioli G., Lauretani F., Davoli M.L., Martini E., Frondini C., Pellicciotti F., Zagatti A., Giordano A., Pedriali I., Nardelli A. (2012). Older People With Hip Fracture and IADL Disability Require Earlier Surgery. J. Gerontol. Ser. A Biol. Sci. Med Sci..

[40] Roberson T.A., Granade C.M., Hunt Q., Griscom J.T., Adams K.J., Momaya A.M., Kwapisz A., Kissenberth M.J., Tolan S.J., Hawkins R.J. (2017). Nonoperative management versus reverse shoulder arthroplasty for treatment of 3- and 4-part proximal humeral fractures in older adults. J. Shoulder Elb. Surg..

[41] Rodrigues A.M., Caetano-Lopes J., Vale A.C., Vidal B., Lopes A., Aleixo I., Polido-Pereira J., Sepriano A., Perpétuo I.P., Monteiro J. (2012). Low osteocalcin/collagen type I bone gene expression ratio is associated with hip fragility fractures. Bone.

[42] Rommens P.M., Hofmann A. (2013). Comprehensive classification of fragility fractures of the pelvic ring: Recommendations for surgical treatment. Inj. Int. J. Care Inj..

[43] Ruggiero C., Zampi E., Rinonapoli G., Baroni M., Serra R., Zengarini E., Baglioni G., Duranti G., Ercolani S., Conti F. (2015). Fracture prevention service to bridge the osteoporosis care gap. Clin. Interv. Aging.

[44] Sabesan V.J., Valikodath T., Childs A., Sharma V.K. (2015). Economic and social impact of upper extremity fragility fractures in elderly patients. Aging Clin. Exp. Res..

[45] Samelson E.J., Demissie S., Cupples L.A., Zhang X.C., Xu H.F., Liu C.T., Boyd S.K., McLean R.R., Broe K.E., Kiel D.P. (2018). Diabetes and Deficits in Cortical Bone Density, Microarchitecture, and Bone Size: Framingham HR-pQCT Study. J. Bone Miner. Res..

[46] Schmitz P., Baumann F., Grechenig S., Gaensslen A., Nerlich M., Muller M.B. (2015). The cement-augmented transiliacal internal fixator (caTIFI): An innovative surgical technique for stabilization of fragility fractures of the pelvis. Inj. Int. J. Care Inj..

[47] Shen S.H., Huang K.C., Tsai Y.H., Yang T.Y., Lee M.S., Ueng S.W.N., Hsu R.W.W. (2014). Risk Analysis for Second Hip Fracture in Patients After Hip Fracture Surgery: A Nationwide Population-Based Study. J. Am. Med. Dir. Assoc..

[48] Sudo H., Ito M., Abumi K., Kotani Y., Takahata M., Hojo Y., Minami A. (2010). One-stage posterior instrumentation surgery for the treatment of osteoporotic vertebral collapse with neurological deficits. Eur. Spine J..

[49] Svensson H.K., Olofsson E.H., Karlsson J., Hansson T., Olsson L.E. (2016). A painful, never ending story: Older women’s experiences of living with an osteoporotic vertebral compression fracture. Osteoporos. Int..

[50] Tanaka S., Narusawa K., Onishi H., Miura M., Hijioka A., Kanazawa Y., Nishida S., Ikeda S., Nakamura T. (2011). Lower osteocalcin and osteopontin contents of the femoral head in hip fracture patients than osteoarthritis patients. Osteoporos. Int..

[51] Tsangari H., Findlay D.M., Zannettino A.C.W., Pan B.Q., Kuliwaba J.S., Fazzalari N.L. (2006). Evidence for reduced bone formation surface relative to bone resorption surface in female femoral fragility fracture patients. Bone.

[52] Westrich G.H., Rana A.J., Terry M.A., Taveras N.A., Kapoor K., Helfet D.L. (2005). Thromboembolic disease prophylaxis in patients with hip fracture—A multimodal approach. J. Orthop. Trauma.

[53] Zhang J., Ang M.L., Kwek E.B.K. (2015). Who Will Walk Again? Effects of Rehabilitation on the Ambulatory Status in Elderly Patients Undergoing Hemiarthroplasty for Femoral Neck Fracture. Geriatr. Orthop. Surg. Rehabil..

[54] Zywiel M.G., Hurley R.T., Perruccio A.V., Hancock-Howard R.L., Coyte P.C., Rampersaud Y.R. (2015). Health Economic Implications of Perioperative Delirium in Older Patients After Surgery for a Fragility Hip Fracture. J. Bone Jt. Surg. Am. Vol..

[55] Kannus P., Parkkari J., Niemi S., Sievänen H. (2015). Low-Trauma Pelvic Fractures in Elderly Finns in 1970–2013. Calcif. Tissue Int..

[56] Bücking B., Neuerburg C., Knobe M., Liener U. (2019). Treatment of patients with fragility fractures. Unfallchirurg.

[57] Fingar K.R., Stocks C., Weiss A.J., Steiner C.A. (2006). Most Frequent Operating Room Procedures Performed in U.S. Hospitals, 2003–2012: Statistical Brief #186. Healthcare Cost and Utilization Project (HCUP) Statistical Briefs.

[58] Lindahl H. (2007). Epidemiology of periprosthetic femur fracture around a total hip arthroplasty. Injury.

[59] Franklin J., Malchau H. (2007). Risk factors for periprosthetic femoral fracture. Injury.

[60] Ensrud K.E. (2013). Epidemiology of fracture risk with advancing age. J. Gerontol. Ser. A Biol. Sci. Med Sci..

[61] Ahmad S.S., Albers C.E., Büchler L., Kohl S., Ahmad S.S., Klenke F., Siebenrock K.A., Beck M. (2016). The hundred most cited publications in orthopaedic hip research—A bibliometric analysis. HIP Int..

[62] Held M., Castelein S., Bruins M.F., Laubscher M., Dunn R., Keel M., Ahmad S., Hoppe S. (2018). Most Influential Literature in Spinal Tuberculosis: A Global Disease Without Global Evidence. Glob. Spine J..

[63] Ioannidis J.P., Boyack K.W., Small H., Sorensen A.A., Klavans R. (2014). Bibliometrics: Is your most cited work your best?. Nature.

